# ChatGPT promotes healthcare: current applications and potential challenges – correspondence

**DOI:** 10.1097/JS9.0000000000001354

**Published:** 2024-03-18

**Authors:** Junting Li, Yuanyuan Bao, Yuanyuan Yang, Changkun Mao

**Affiliations:** a Department of Urology, Anhui Provincial Children’s Hospital; b Department of Electrocardiogram, Anhui Maternal and Child Health Hospital, Hefei, Anhui, People’s Republic of China

*Dear Editor,*

Just as the invention of the steam engine in 17th-century Britain propelled the Industrial Revolution and unprecedented development in productivity, the emergence of artificial intelligence (AI) in the 21st century has ushered in a new era of technological revolution^1^. Since its development by OpenAI in November 2022, ChatGPT has been widely used and discussed across various industries globally, becoming one of the hottest topics today^2^. We were particularly interested in reading the article by Wu and Zhang^3^, which explores the application of ChatGPT in the medical field and the challenges it faces from multiple dimensions. The article provides a detailed discussion of ChatGPT’s functions and potential, showcasing its applications in scientific research, health consultation, medical education, and more, while also highlighting the ethical, privacy, and data security challenges involved. We commend the authors for their achievements in this field and wish to share our views to further enrich the discussion on this topic.

Firstly, although the article offers a comprehensive overview of the potential applications of ChatGPT in healthcare, it lacks an in-depth analysis of specific application cases. Introducing actual cases of ChatGPT’s use in diagnosing specific diseases, patient consultation, or medical data analysis not only provides readers with a more concrete and vivid understanding but also helps assess the technology’s effectiveness and feasibility in real medical environments^4^. It has been reported that ChatGPT is widely discussed and used in medical fields such as urology and psychiatry^5,6^, where it contributes to enhancing the work experience of healthcare professionals. Moreover, case analyses can demonstrate how ChatGPT solves specific medical problems, such as improving diagnostic accuracy, creating personalized treatment plans, and optimizing the allocation of medical resources. Through the analysis of successful cases and challenges, researchers and practitioners can gain valuable insights to guide future technological improvements and application strategies. Given that healthcare is a multidisciplinary field involving medicine, psychology, information technology, and more, case studies should also explore collaborations between healthcare professionals and computer scientists, and how such interdisciplinary cooperation can promote technological innovation and application^6,7^. We encourage future researchers to delve deeper into and publish more case studies on ChatGPT’s applications in healthcare to enrich research content, promote knowledge sharing, and advance technological progress.

Secondly, for healthcare applications, designing an intuitive and easy-to-navigate user interface is crucial. This includes clear instruction prompts, easy-to-understand menu layouts, and user-friendly interaction designs for non-technical users. Ensuring that all users, including those with visual, hearing, or other disabilities, can effectively use ChatGPT is another important aspect of enhancing user experience^6^. Advanced AI technologies, like ChatGPT, can play a pivotal role in this by leveraging user data to personalize interfaces and interactions dynamically. Moreover, the integration of inclusive design principles ensures that digital health tools are accessible to everyone, thereby reducing barriers to healthcare access^8^. Additionally, integrating an effective user feedback mechanism allows users to report issues, suggest improvements, or share their experiences. This feedback mechanism should go beyond traditional methods, incorporating real-time analytics to predict user needs and preferences, thereby fostering more proactive user engagement and system improvements. This not only aids in continuously enhancing the performance and user interface of ChatGPT but also boosts user participation and satisfaction with the system improvement process.

Overall, the research by Wu and Zhang provides us with deep insights into the application of ChatGPT in the healthcare field, while also pointing out the directions for future research and practice. We look forward to more such studies, not only exploring the potential of ChatGPT but also offering concrete strategies to address the practical challenges of its application.

## Ethical approval

Dear editor, as this article is a correspondence about ChatGPT, there is no need for approval from the ethics committee.

## Consent

This manuscript does not require it.

## Sources of funding

None.

## Authors contribution

J.L. and Y.B.: conceptualization and writing; Y.Y. and C.M.: review, editing, and supervision.

## Conflicts of interest disclosure

The authors declare no conflicts of interest.

## Research registration unique identifying number (UIN)

Not applicable.

## Guarantor

All authors.

## Data availability statement

Not applicable.

## Provenance and peer review

Yes, we agree.
